# Enhancing Nutraceutical Quality and Antioxidant Activity in Chili Pepper (*Capsicum annuum* L.) Fruit by Foliar Application of Green-Synthesized ZnO Nanoparticles (ZnONPs)

**DOI:** 10.3390/nano15181440

**Published:** 2025-09-18

**Authors:** Daniela Monserrat Sánchez-Pérez, Jolanta E. Marszalek, Jorge Armando Meza-Velázquez, David Francisco Lafuente-Rincon, Maria Teresa Salazar-Ramírez, Selenne Yuridia Márquez-Guerrero, Maria Guadalupe Pineda-Escareño, Agustina Ramírez Moreno, Erika Flores-Loyola

**Affiliations:** 1 Facultad de Ciencias Biológicas, Universidad Autónoma de Coahuila, Torreón 27276, Mexico; monserrat.sanchez@uadec.edu.mx (D.M.S.-P.); j.marszalek@uadec.edu.mx (J.E.M.); david_lafuente@uadec.edu.mx (D.F.L.-R.); salazar.teresa@uadec.edu.mx (M.T.S.-R.); mapineda@uadec.edu.mx (M.G.P.-E.); agustina-ramirez@uadec.edu.mx (A.R.M.); 2 Facultad de Ciencias Químicas, Universidad Juárez del Estado de Durango, Av. artículo 123 s/n, Gómez Palacio, Durango 35020, Mexico; jorgemezav68@gmail.com; 3 Programa Agua-Suelo, Tecnológico Nacional de México, División de Estudios de Posgrado e Investigación, Instituto Tecnológico de Torreón, Torreón 27190, Mexico; selenne.mg@torreon.tecnm.mx

**Keywords:** enzymes, bio-zinc oxide nanoparticles, antioxidants, nutraceutical quality, chili peppers

## Abstract

The application of zinc oxide nanoparticles prepared by green synthesis (GS-ZnONPs) has demonstrated essential benefits in boosting the clean and sustainable production of agricultural crops worldwide. In this part of the study we evaluate the effect of GS-ZnONPs foliar spraying on the yield, nutraceutical quality, capsaicin concentration, and antioxidant metabolism of chili fruit (*Capsicum annuum* L., CHISER-522 variety) grown under greenhouse conditions. GS-ZnONPs treatments were applied at concentrations of 10, 20, 30, 40, and 50 ppm every 15 days post-transplant, with the control group treated only with distilled water. The results indicated that treatments with 40 and 50 ppm of GS-ZnONPs significantly improved fruit yield, length, and fruit amount. At the same time, the concentrations of 30 and 40 ppm significantly increased the levels of vitamin C, bioactive compounds, and antioxidant capacity, indicating a better nutraceutical quality of the fruit. In addition, an increase in the catalase activity and the content of macro and micro-minerals in the fruit treated with GS-ZnONPs was observed. Our results suggest that the foliar application of GS-ZnONPs acts as a nanobioestimulant, offering an excellent biotechnological tool for developing agroecological strategies to increase the nutraceutical and antioxidant quality of chili pepper fruit.

## 1. Introduction

The ever-increasing demand for foods with enhanced nutraceutical and antioxidant properties is strongly present among the public. This type of food promotes consumers’ physical and mental well-being [1,2]. In this context, the fruit of chili peppers (*Capsicum annuum* L.), one of the world’s leading gastronomic ingredients [3], has gained importance as an agricultural crop. The chilies contain a rich amount of bioactive compounds, such as phenolic antioxidants and capsaicinoids, which contribute to their characteristic spicy flavor [4]. Antioxidant compounds play a crucial role in chili peppers’ nutraceutical properties, ensuring protection against oxidative stress that can contribute to the prevention and treatment of various diseases [5,6]. There is a growing interest in reducing the environmental impact of agricultural crop production (as chili) and at the same time improving the product’s nutraceutical and antioxidant properties. One such innovative approach is the use of nanomaterials [7,8], especially those developed through green synthesis. Nanofertilizers enhance the properties of conventional treatments by reducing the environmental impact of agricultural production. Bio-stimulants, in the form of nanoparticles such as zinc oxide nanoparticles, exhibit a significant increase in contact area thanks to their small size (defined as below 100 nm). In turn, a large surface area improves micronutrient interaction and nutrient uptake [9]. The properties of the nanoparticles are influenced by their size, shape, chemical composition, physicochemical stability, crystalline structure, surface area, surface energy, and the presence or absence of active compounds on their surface [10]. These characteristics are determined by the method used to obtain the nanoparticles. The green synthesis, which utilizes biological extracts, produces materials that are most biocompatible with plants, as they contain a thin layer of active compounds. That bio-layer improves their absorption and delivery [11]. Furthermore, it has been proven that green-synthesized ZnONP at low concentration causes less phytotoxicity than those chemically synthesized [12], hence the importance of using green synthesis to obtain these NPs.

The foliar application of nanomaterials promotes plant growth, allowing for a controlled, precise release and uniform distribution of the stimulant within plant tissues. This form of nanofertilizer application is beneficial since it gradually and precisely provides nutrients to plants [13].

For the ultimate growth and development of plants, zinc is a key micronutrient, as it participates in various metabolic processes, including chlorophyll formation [13], photosynthesis, protein synthesis [14], and the regulation of phytohormones [14]. In addition to being a direct source of zinc, zinc oxide nanoparticles (ZnONPs) have emerged as a tool in modern agriculture thanks to their unique ability to act as plant biostimulants [15,16]. ZnONPs increase the activity of antioxidant enzymes by acting as cofactors, which helps to eliminate reactive oxygen species and protects plant cells against oxidative stress [17]. ZnONPs can also modify the gene expression related to plant growth, specifically those involved in oxidative stress, and increase the synthesis of phytohormones, such as auxins, cytokinins, and gibberellins [18]. Phytohormones regulate essential processes that contribute to plant vigor, including cell division, stem elongation, and root formation. Additionally, ZnONPs can enhance the photosynthetic efficiency of plants by optimizing the conversion of light into chemical energy. They cause changes in the physical, chemical, and biological characteristics of plants. This is because zinc regulates auxins [19], protein metabolism, carbohydrate biosynthesis, and protects plants from environmental stress [20]. Consequently, these influences affect chemical and biological activities that induce oxidative stress and toxicity in plants, while triggering their antioxidant production systems [21]. Previously, our research group observed positive effects of GS-ZnONPs in the *Capsicum annuum* seed germination process, in which physiological variables such as seed germination and seedling vigor were improved [22]. However, ZnONPs are also involved in crop yields, as shown by others [23]. They enhance the nutraceutical quality of fruit obtained from plants by increasing their phytochemical compounds’ levels [13,24]. This process starts by generating stress in the plants, which increases the concentration of reactive oxygen species (ROS), thereby activating both the enzymatic and non-enzymatic defense systems [25]. Subsequently, the concentration of enzymes, such as catalase, also increases [26]. This was demonstrated by Faizan et al. in 2020, who found that soaking tomato plant roots in 10 ppm ZnONPs concentrations for 30 min significantly increased bioactive compounds levels and antioxidant enzymes activity [27]. These parameters have gained importance in recent years because the content of phenolic compounds is considered one of the most critical nutraceutical value parameters in chili fruit [13]. Consumers are more likely to accept fruits with naturally occurring antioxidants than those with synthetic ones, making products with high phytochemical levels of greater commercial interest [26].

Although the effect of ZnONP on different crops has been reported [28,29,30] only a few of those have focused on the nutraceutical and antioxidant qualities of fruits from specific *Capsicum annuum* varieties. Our work provides novel evidence by evaluating the chili fruit of the CHISER-522 variety, which has productive and commercial relevance. We evaluate various aspects of the fruit from plants treated with foliar applications of nanoparticles synthesized through green methods at a range of low, ecologically viable concentrations. This approach integrates sustainability and nutraceutical quality of the fruit, an aspect that is little explored in the existing literature.

## 2. Materials and Methods

The experiments were conducted over three spring–summer cycles (March to June 2022, 2023, and 2024) at the Technological Institute of Torreon (25°36′37″ North, 103°22′33″ West, at an altitude of 1150 m above sea level), located at kilometer. 7.5 of the old Torreon-San Pedro Highway, Municipality of Torreon, Coahuila, Mexico. Zinc oxide nanoparticles (ZnONPs) used in this study were synthesized using a green synthesis method previously reported by our research team [23], for which detailed characterization was carried out. This characterization included SEM, XRD, zeta potential, XPS, and size distribution analysis, confirming the nanoparticle size to be 20–40 nm and presenting a stable crystalline structure.

### 2.1. Plant Material

Seeds of CHISER-522 soledad pepper were donated by the Regional Research Center of the Northeast (INIFAP), Las Huastecas experimental field. These seeds were sown in a polystyrene germination tray with 200 spaces, using an organic peat moss substrate (Premier Sphagnum Peat Moss). Transplanting was carried out into plastic pots, 20 cm in diameter, with a capacity of 6 kg, 17 days after planting, when the seedlings had at least 2 true leaves and an average height of 5 cm. A mixture of peat moss, sand, and perlite was used as a seeding substrate (40:40:20 *v*/*v*). For each treatment, 10 plants (biological replicates) were grown per year for three consecutive years (2022–2024), totaling 30 plants per treatment. During the development of the crop, Steiner’s nutrient solution was applied [31]. The pots were kept at field capacity in a mixed greenhouse with low brick walls, casement windows, and plastic cover with shade mesh. The day temperatures oscillated between 22 and 24 °C, and at night, between 17 and 20 °C. The relative humidity was maintained at 65–75% and a radiation of 700–800 W/h.

### 2.2. Application of Nanoparticles

Foliar applications of GS-ZnONPs were performed every 15 days, starting 20 days after transplanting. Each treatment consisted of 200 mL of nanoparticle suspension per plant, prepared in deionized water and homogenized using a probe sonicator (Fisher Scientific, Hampton, NH, USA) at specific cycles and power for 30 min prior to application. Treatments included five concentrations (10, 20, 30, 40, and 50 ppm) of GS-ZnONPs and a control treated with distilled water. The reported concentrations correspond to the total ZnO content present in the nanoparticle suspension, not just the elemental Zn content. All analyses were conducted on fruit at harvest (120 days after transplant).

### 2.3. Fruit Yield Assessment

At the time of harvest (140 days after transplanting), 120 fruits were collected from each plant and used in the analyses. All fruits were washed, weighed, and counted to determine yield parameters, including fruit length and diameter, number of fruits per plant, average fruit weight, and total fresh weight per plant.

### 2.4. Vitamin C Content

Vitamin C content was determined by titration [32]. 10 g of fruit (seedless parts) were ground with 10 mL of 2% hydrochloric acid, filtered, and the filtrate was diluted to 100 mL with distilled water. A 10 mL aliquot of the extract was titrated with 2,6-dichlorophenolindophenol (1 × 10^−3^ Eq-g-L^−1^), and results were expressed as mg of vitamin C per 100 g of fresh sample.

### 2.5. Chlorophyll and Carotenoid Content

Total chlorophyll and carotenoid content was determined using Lichtenthaler’s method [33]. 1 g of fresh fruit (excluding seeds) was ground in 5 mL of pure acetone, filtered, and diluted to 10 mL with the same solvent. Absorbance readings were taken at 665 and 649 nm using a Jenway 7305 UV-Vis spectrophotometer. Chlorophyll content was calculated using Lichtenthaler’s equations. Chlorophylla a = 11.24A_662_ − 2.04A_645_, Chlorophyll b = 20.13A_645_ − 4.19A_662_, Total Chlorophyll = 7.05A_662_ + 18.08A_645_, Carotenid = (1000A_640_ − 1.9C_a_ − 63.14C_b_)/214 [33].

### 2.6. Bioactive Compounds

Extract Preparation: 1 g of fresh sample (seedless fruit) was ground in 10 mL of 80% aqueous methanol and agitated at 70 rpm for 24 h at room temperature. The extract was centrifuged at 5000 rpm for 5 min, and the supernatant was used for subsequent analyses [34].

Total Phenolic Content (TPC) was quantified using the Folin–Ciocalteu method [35]. 300 µL of the extract was mixed with 1080 µL of deionized water, 120 µL of Folin–Ciocalteu reagent, and vortexed for 10 s. After 10 min, 900 µL of 7.5% Na_2_CO_3_ was added, and the mixture was vortexed again. Samples were kept at room temperature for 30 min, and absorbance was measured at 765 nm using a Jenway 7305 UV-Vis spectrophotometer. Results were expressed as mg of gallic acid equivalents per 100 g of fresh weight (mg GA 100 g^−1^ FW).

Total Flavonoid Content was determined using the aluminum chloride colorimetric assay [36]. 250 µL of the extract was combined with 1.25 mL of DI water and 75 µL of 5% NaNO_2_. After 5 min, 150 µL of 10% AlCl_3_ was added. Six minutes later, 500 µL of 1 M NaOH and 275 µL of deionized water were added, and absorbance was measured at 510 nm using a Jenway 7305 UV-Vis spectrophotometer. Results were expressed as mg of catechin per 100 g of fresh weight.

Antioxidant Activity was assessed using the DPPH assay [37]. 50 µL of the extract was mixed with 1950 µL of 0.025 mg mL^−1^ DPPH solution in ethanol. Absorbance was read at 517 nm after 30 min using Jenway 7305 UV-Vis spectrophotometer. Results were expressed as milliequivalents of Trolox per 100 g of fresh weight.

### 2.7. Capsaicin and Dihydrocapsaicin Content

The content of capsaicin and dihydrocapsaicin was determined by the Collins method [38]. The fruits were harvested, washed, and immediately frozen after cutting, then ground and freeze-dried. To obtain the extract [39] 0.5 g of dried fruit was mixed with 5 mL of pure acetone (J.T. Baker) for 4 h at 50 °C at 400 rpm. The mixture was centrifuged (Thermo Fisher Scientific, Waltham, MA, USA) for 10 min at 5000 rpm, the extract obtained was filtered through cellulose filters with 0.2 micron pores (Advangene, Lake Bluff, IL, USA), 20 μL of the filtered extract was injected into the chromatograph. The chromatographic conditions were as follows: Agilent column C8 (particle size 5 μm, dimension 150 × 4.6 mm), mobile phase was a water–acetonitrile mixture in a ratio of 60:40, and flow rate 1 mL/min. The pattern was prepared with standard solutions of capsaicin and dihydrocapsaicin using series dilutions 25–200 ppm under the same chromatographic conditions, and the results were reported as mg of capsaicin kg^−1^ dry chili mass.

### 2.8. Enzyme Activity of Catalase

The extract for the enzymatic activity of the catalase enzyme was prepared using 1 g of fresh fruit, ground in 10 mL of 100 mM phosphate buffer (pH 6.8) at 4 °C. The extract was centrifuged at 5000 rpm for 15 min at 4 °C, and the enzyme analysis was performed on the supernatant.

Catalase enzyme activity (CAT 1.11.1.6) was measured according to the Aebi method [40]. CAT activity was measured spectrophotometrically (Jenway 7305 UV-Vis, Sheung Wan, Hong Kong) at room temperature by controlling the decrease in absorbance at 240 nm resulting from the decomposition of H_2_O_2_. The molar extinction coefficient was used (ε240 = 43.6 M^−1^ cm^−1^) [41] and protein content [42] to calculate enzyme activity. The activity was expressed in U mg^−1^ protein, where one unit (U) of catalase activity was defined as the amount of enzyme that caused an absorbance change of 0.001 per minute in the assay conditions.

### 2.9. Nitrogen and Mineral Content in Chili Fruit

For nitrogen (N) determination, fruit samples were digested using the Kjeldahl method [43], which involves the transformation of organic N to ammonium (NH_4_) by digesting the sample with concentrated sulfuric acid (H_2_SO_4_) and then measuring the amount of NH_4_ produced. Nitrogen concentration was expressed as g kg^−1^. Phosphorus (P) was determined by the colorimetric method of ammonium metavanadate (NH_4_VO_3_) in an absorption at 430 nm against a K_2_HPO_4_ curve. In total, 3.5 mL of distilled water, 500 μL of the ammonium metavanadate (NH_4_VO_3_) stock solution, and 1 mL of phosphorus reagent were added to the test tubes. Each tube was shaken in a vortex and left to stand for an hour. At the end, the reading was measured spectrophotometrically (Jenway 7305 UV-Vis, Sheung Wan, Hong Kong). Phosphorus concentration was expressed as g kg^−1^. The total contents of K^+1^, Ca^+2^, Mg^+2^, Cu^+2^, Fe^+2^, Zn^+2^, and Mn^+2^ were determined after sample digestion with 65% nitric acid. Dry fruit samples were weighed in digestion tubes, and 10 mL of nitric acid was added. The tubes were heated in an infrared digestion device (Behr Labor-Technik™ B00218105, Düsseldorf, Germany). Nitric acid was added as needed to complete digestion. The solution was allowed to dry when the content of the tubes was clear. The residue was dissolved in a mixture of sufficient nitric acid and lanthanum solution to achieve a final concentration of 1% HNO_3_ + 0.5% lanthanum (99.99%) when the solution was brought to the volume of the flask used. Then, the solution obtained was used to determine the concentrations of potassium, calcium, magnesium, copper, iron, zinc, and manganese using atomic flame absorption spectrometry (F-AAS) with an ICE from Thermo Scientific. Targets and calibration standards were read for quality control purposes. The results were expressed as g and mg of dry weight kg^−1^ of each element [44].

### 2.10. Statistical Analysis

Data were analyzed considering each treatment as a fixed effect and the crop year as a random effect using a linear mixed model (LMM). Additionally, a two-way ANOVA with treatment and crop year factors and interaction (treatment × crop year) was performed to corroborate results. Normality of residuals (Shapiro–Wilk) and homogeneity of variances (Levene) were verified by applying transformations (log_10_ or Box–Cox) when necessary. Comparisons were made using Tukey’s HSD test (*p* ≤ 0.05). All results represent means of three replicates. All analyses were performed using SAS version 9.4.

## 3. Results

### 3.1. Fruit Yield

Table 1 presents the effect of different concentrations of zinc oxide nanoparticles (GS-ZnONPs) on fruit yield in terms of diameter, length, fresh weight, number of fruits per plant, and total fruit weight. Significant differences were observed in all evaluated variables (*p* ≤ 0.05). Plants with application of 40 and 50 ppm of GS-ZnONPs via foliar application produced a higher number of fruits, with increases of 34% and 31%, respectively, compared to the control treatment. The highest average fruit weight was recorded with applications of 30 and 40 ppm of GS-ZnONPs, exceeding the control by 28% and 29%, respectively. Regarding fruit length, plants treated with 40 and 50 ppm of GS-ZnONPs showed increases of 38% and 37%, respectively, compared to the control. The diameter of the fruit showed a highly significant increase with the treatment of 40 ppm of GS-ZnONPs, with a 54% increase compared to the control. Similarly, total fruit weight was higher in plants treated with 40 ppm GS-ZnONPs, outperforming the control by 61%.

### 3.2. Vitamin C Content

The levels of vitamin C in chili fruit (Table 2) significantly increased upon the GS-ZnONPs foliar application. The highest concentration was measured at 40 ppm, representing an 18% increase compared to the control. The results suggest that vitamin C in the fruit increases with moderate concentrations of GS-ZnONPs. However, in plants treated with 50 ppm of GS-ZnONPs, a 12% decrease in the fruit vitamin C content is observed compared to the control.

### 3.3. Bioactive Compounds

#### 3.3.1. Total Phenol Content

**Figure 1:**
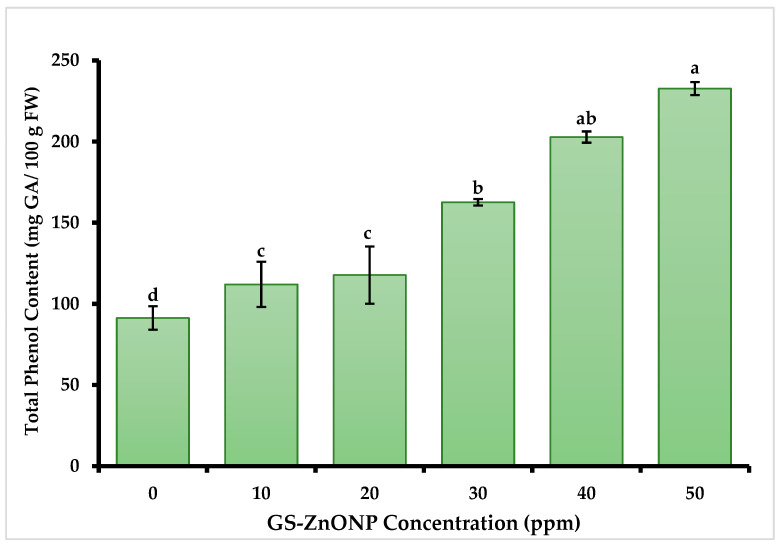
Effect of GS-ZnONPs treatments at different concentrations (0 ppm indicates control without treatment) on the Total Phenols Content in the fruit of *Capsicum annuum*. Values are the average of 3 measurements ± SD. Values with different letters indicate a significant difference, according to Tukey’s test (*p* ≤ 0.05).

The results indicate that foliar application of GS-ZnONPs significantly affected the content of total phenols and flavonoids in the chili fruit. The highest TPC (Figure 1) was measured in the 30 and 40 ppm treatments, with contents of 202.7 ± 3.4 and 232.7 ± 3.9 mg GA 100 g FW^−1^, respectively, 121% and 154% higher than the control treatment.

#### 3.3.2. Total Flavonoids

**Figure 2:**
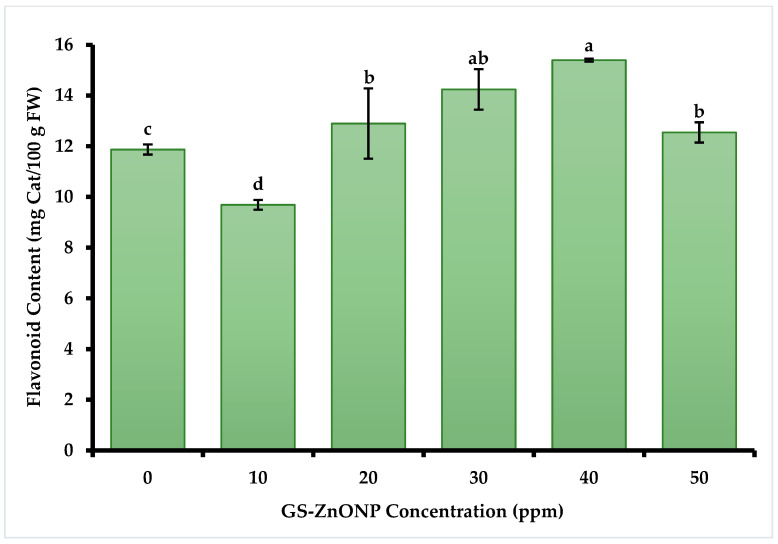
The flavonoid content in *Capsicum annuum* fruit treated with different GS-ZnONP concentrations (0 ppm indicates untreated control). Values are the average of 3 measurements ± SD. Values with different letters indicate a significant difference, according to Tukey’s test (*p* ≤ 0.05).

Similar effects were observed in flavonoid concentration (Figure 2). The highest concentration of total flavonoids was found in plants treated with 40 ppm of GS-ZnONPs, with a concentration of 15.39 ± 0.80 mg Cat 100 g FW^−1^.

#### 3.3.3. Antioxidant Capacity

**Figure 3:**
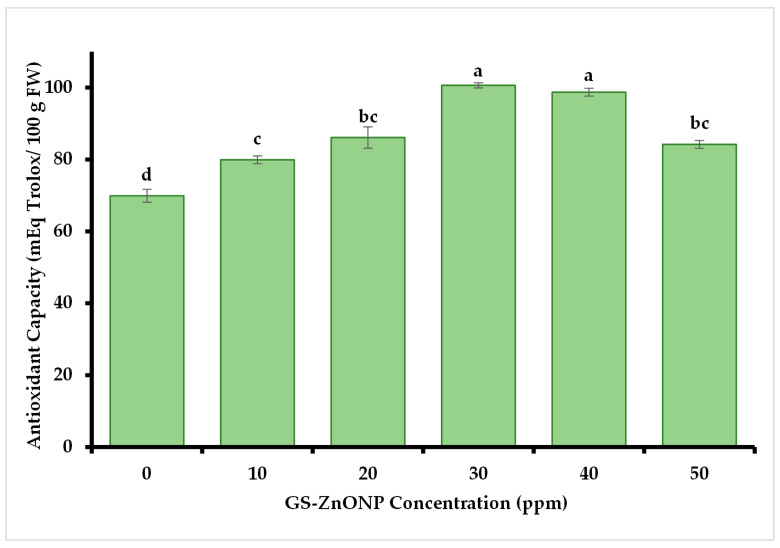
The antioxidant capacity of *Capsicum annuum* fruit treated with different GS-ZnONP concentrations (0 ppm indicates untreated control). Values are the average of 3 measurements ± SD. Values with different letters indicate a significant difference, according to Tukey’s test (*p* ≤ 0.05).

The results for antioxidant capacity (Figure 3) show a significant difference with greater capacity in the fruits obtained from plants treated with 30 and 40 ppm, at 100.61 ± 2.9 and 98.7 ± 0.7 mEq Trolox 100 g FW^−1^, respectively.

### 3.4. The Concentration of Capsaicin and Dihydrocapsaicin

**Figure 4:**
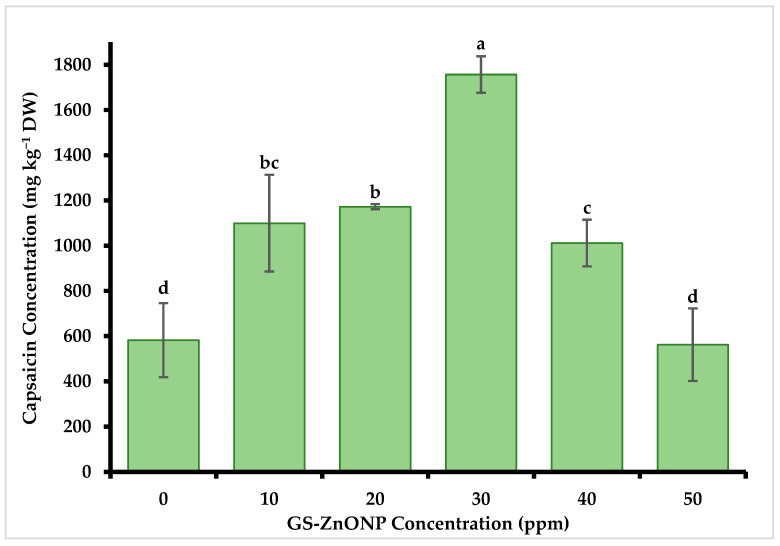
Capsaicin concentration in *Capsicum annuum* fruit treated with different GS-ZnONP concentrations (0 ppm indicates untreated control). Values are the average of 3 measurements ± SD. Values with different letters indicate a significant difference, according to Tukey’s test (*p* ≤ 0.05).

**Figure 5:**
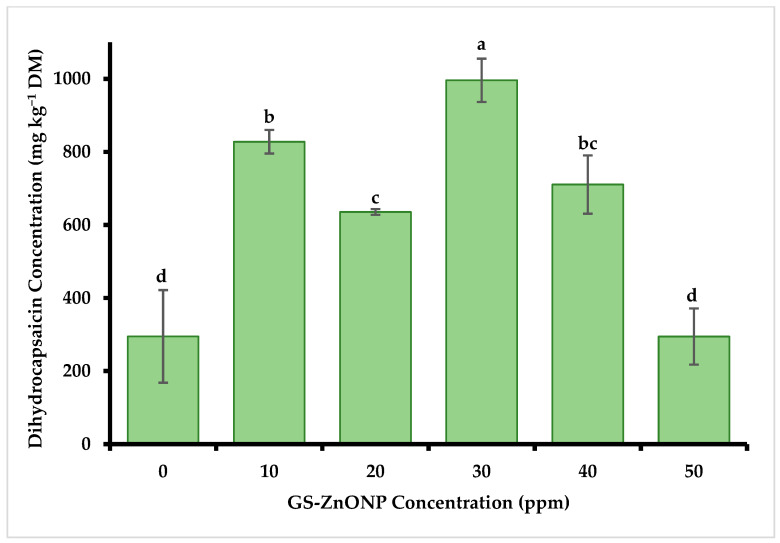
Dihydrocapsaicin concentration in *Capsicum annuum* fruit treated with different GS-ZnONP concentrations (0 ppm indicates untreated control). Values are the average of 3 measurements ± SD. Values with different letters indicate a significant difference, according to Tukey’s test (*p* ≤ 0.05).

The results indicated that capsaicin content was significantly affected by the application of foliar fertilization with GS-ZnONPs (Figure 4). The highest accumulation was detected in plants treated with 30 ppm of GS-ZnONP with a concentration of 1756.90 ± 11.10 mg kg^−1^, which was 200% higher than the control treatment (582.60 ± 160.73 mg kg^−1^). The same trend was observed in dihydrocapsaicin content (Figure 5). Treatment with 30 ppm of GS-ZnONPs resulted in the most significant increase, 70% (996.16 ± 7.81 mg kg^−1^), compared to the control (294.97 ± 160.80 mg kg^−1^). In contrast, the treatment with 50 ppm was statistically equal to the control for both compounds.

### 3.5. Catalase

**Figure 6:**
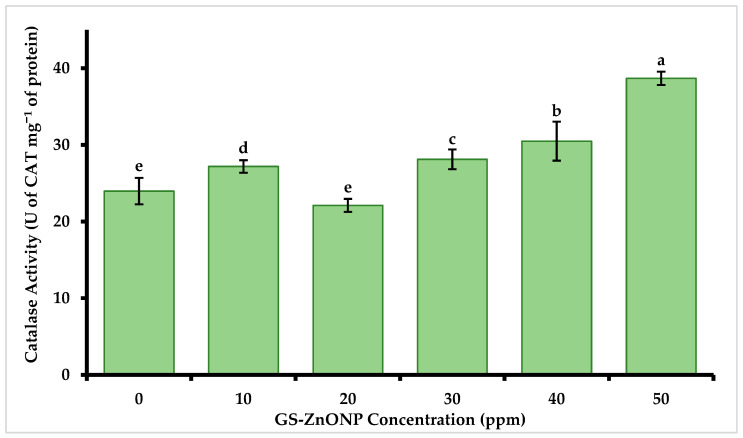
Effect of GS-ZnONP treatments at different concentrations (0 ppm indicates untreated control) on the enzymatic activity of catalase in *Capsicum annuum* fruit. Values are the average of 3 measurements ± SD. Values with different letters indicate a significant difference, according to Tukey’s test (*p* ≤ 0.05).

The enzymatic activity of catalase from chili fruits showed significant differences under the treatment with the biosynthesized GS-ZnONPs (Figure 6). The fruits obtained under the foliar application of 50 ppm of GS-ZnONPs showed a 61% increase (38.68 ± 2.22 U of CAT mg^−1^ of protein) with respect to the control.

### 3.6. Nitrogen and Minerals in Fruit

Significant differences were observed in the content of macro minerals under the nanoparticle treatments (Table 3). All GS-ZnONP treatments increase nitrogen levels in plants; however, plants sprayed with 30 ppm of GS-ZnONPs showed a 25% increase in N concentration compared to the control. Phosphorus levels were the highest in plants treated with 20 and 30 ppm, with increases of 30% and 29%, respectively, compared to the control. Calcium levels were the least varied under the treatments, yet more significant than in the control. Magnesium and sodium levels increased with higher GS-ZnONP concentrations. However, the highest concentration of potassium was evaluated at 10 ppm of nanoparticles.

Slightly different trends can be seen in the microminerals content (Table 4), where copper concentrations are the highest under 20 and 30 ppm treatments, and iron, manganese, and zinc reach higher levels at higher GS-ZnONP treatments.

## 4. Discussion

The ZnONPs have gained importance in agriculture due to their multiple benefits, mainly the large surface area of the small-sized nanoparticles, which impacts mobility and surface activity [45]. This study found that GS-ZnONPs improved the quality and yield of chili fruit, resulting in increased size (diameter and length), weight, and number of fruits when treated with a concentration of 40 ppm. The explanation can be based on the fact that the foliar application of GS-ZnONPs possibly favors greater absorption through the leaves [46]. This directly impacts plant growth and increases biomass production [47], resulting in a larger number of fruit, as seen in this study. As an essential micronutrient, zinc, is crucial for the vegetative development of plants [48]. Zinc acts as a precursor to the production of auxins, regulators of plant growth that influence cell elongation and division. It participates in the synthesis of proteins and photosynthesis processes, leading to higher levels of photoassimilates and larger biomass [49]. Our results show that chili treatment with 30 to 40 ppm of GS-ZnONPs increases these parameters. These findings are consistent with those reported by Ahmed et al. in 2023, who observed increased tomato yield with the application of 100 ppm of ZnONPs [50]. However, one aspect to consider is that the effects observed in this study do not allow for a clear distinction between whether the plant response came exclusively from the applied nanoparticles or from Zn^2+^ ions released by the partial dissolution of ZnO. This has been discussed in other reports [10,22]. However, it has been suggested that ZnONP exert additional effects beyond ionic nutrition, associated with their size, surface area, and ability to induce specific physiological responses in plants [51].

We recorded a decrease in certain parameters, such as fruit weight, at the 50 ppm application. At higher ZnONP levels, there is a possibility of zinc and nanoparticle accumulation leading to the phytotoxic effect [52,53], which causes the increase in osmotic pressure. In turn, it forces ZnONPs to penetrate cell walls and be adsorbed by membranes, resulting in mechanical damage to the cell structure [54]. The observed phytotoxic effects, such as growth reduction and physiological alterations, could be related to the overaccumulation of Zn and the induced oxidative stress, as mentioned in previous studies in other species [52,53]. It should be noted that although the presence of ZnONPs in fruits was not directly measured, previous studies suggest that foliar translocation of nanoparticles to edible tissues is generally limited [54]. Furthermore, the partial transformation of ionic forms of Zn [10,55] during plant metabolism reduces the accumulation of intact particles, keeping zinc levels within safe ranges for human consumption. Our results show increased content of Zn^+2^ under all treatments (up to 123.12 ± 1.02 mg/Kg), as expected. Yet, they are safe for consumption, with the recommended daily intake of 40 mg of Zn [56]. Nevertheless, direct analysis of nanoparticles in fruits would be useful for a comprehensive assessment of food safety.

We have observed an 18% increase in the concentration of ascorbic acid in the chili fruit. The induced oxidative stress can cause the vitamin C level to increase with the higher ZnONP concentration, since ascorbic acid acts as an oxidant and cofactor in the biosynthesis of other antioxidants [57]. This increase raises the nutritional value of the fruit because vitamin C is essential for humans [58] and must be obtained through diet [32,59]. However, when ZnONP concentration is as high as 50 ppm, this value decreases, probably due to the inhibition of the enzyme activity of ascorbic acid metabolism produced by the effect of metal ions [26].

At the GS-ZnONPs concentrations of 30 to 50 ppm, we observed a significant increase in phenols and flavonoids concentrations, which suggests that ZnONPs induce an increased production of bioactive compounds in *Capsicum annuum* plants, possibly due to the expression of genes responsible for the biosynthesis of phenolic compounds [60]. Plants produce antioxidants such as phenols, carotenoids, and antioxidant enzymes as part of a protective mechanism to limit oxidative damage caused by ROS [61]. Phenolic compounds, as electron donors in organelle structures, work to detoxify ROS [62]. Thanks to their redox characteristics, they directly eliminate active oxygen species. In addition, phenolic compounds participate in the absorption and neutralization of free radicals and the decomposition of peroxides [63]. Since ZnO nanoparticles applied to plants cause abiotic stress [64], a greater phenol and flavonoid accumulation in the fruits of plants exposed to treatments with ZnONPs can be observed, as seen in this study. Our results fully agree with that explanation. We noted a 151% increase in total phenolic content, a 30% increase in flavonoid levels, and a 43% increase in antioxidant activity in chili fruit when the plants were treated with a 40 ppm GS-ZnONPs suspension. Similar effects have been previously reported in jalapeño and serrano peppers [65,66]. Although ROS were not directly measured here, the activation of antioxidant enzymes and the accumulation of bioactive compounds constitute reliable indirect markers of induced oxidative stress, supporting the proposed mechanism of ZnONPs’ action [67].

Additionally, the substantial catalase activity increase observed in this study reinforces the role of GS-ZnONPs in modulating ROS homeostasis. This effect may be explained by a dual mechanism: (i) the release of Zn^2+^ ions, which act as cofactors for antioxidant enzymes, directly enhancing catalase activity [45]; and (ii) the induction of mild oxidative stress by the nanoparticles, leading to transient ROS accumulation that activates redox signaling pathways [29]. These ROS molecules, especially H_2_O_2_, act as secondary messengers that regulate the expression of defense-related genes and stimulate the biosynthesis of antioxidant metabolites. Thus, the enhanced catalase activity in *Capsicum* reflects a balance between ROS production and antioxidant induction, which contributes to maintaining redox homeostasis and supports the increased accumulation of phenols, flavonoids, and capsaicinoids.

The antioxidant capacity of the fruit is related to the levels of capsaicinoids, which are recognized antioxidants in chili fruit [65] and have shown protective functions against ROS [4]. Therefore, the observed substantial increase (160% in capsaicin and 230% in dihydrocapsaicin under 30 ppm ZnNPs treatment) may be due to oxidative stress caused by the presence of ZnONPs. The metallic NPs alter the concentrations of Ca^2+^ and ROS involved in cell signaling and the complex physiological and biochemical functions of the plants [68]. In our case, the plant’s defense system accumulated higher concentrations of enzyme and non-enzyme antioxidant compounds, resulting in a higher accumulation of capsaicinoids (Figure 4 and Figure 5). This suggests that moderate doses of GS-ZnONPs favor the biosynthesis of these metabolites, whereas higher concentrations may trigger partial metabolic saturation or early phytotoxic effects [69], limiting further accumulation [51]. Overall, GS-ZnONPs enhance foliar zinc absorption, stimulate growth and the accumulation of bioactive compounds, and modulate oxidative stress by activating antioxidant enzymes, thereby improving fruit quality and yield.

On the other hand, the effect of the ZnO nanoparticles mentioned above was confirmed by evaluating the mineral content in the chili fruits. The total concentration of Cu^2+^, Fe^2+^, Zn^2+^, and Mn^2+^ showed a significant increase compared to the control treatment. Additionally, our results showed an increase in nitrogen absorption. Zn is related to N metabolism in the plant, and it correlates with the activity of the enzyme nitrate reductase [70], so the higher concentration of zinc in plants increases the nitrogen concentration. It has been shown that the deficiency or toxicity of Zn inhibits the enzyme nitrate reductase, which leads to a decrease in N content and a decrease in the incorporation of N into amino acids and proteins [71]. Phosphorus is a structural element in nucleic acids, playing a key role in energy transfer as a component of adenosine triphosphates, and is essential for carbohydrate transfer in leaf cells. K^+^ affects sucrose load and solute movement rate driven by mass flow within the plant. Ca^2+^ is essential for cell wall and membrane stabilization, as well as osmoregulation. As a second messenger, it enables plants to regulate development processes in response to environmental stimuli. Mg^2+^ is a chlorophyll component necessary for photosynthesis and protein synthesis [72]. Applying ZnONP suspensions to growing plants resulted in increased levels of these elements in the tissue, thereby improving plant growth and function. This led to increased fruit production and mass (up 34% and 61%, respectively, at a 40 ppm concentration).

In summary, the foliar applications of ZnONPs at 40 and 50 ppm increased the number of fruits, fruit length, and fruit diameter, which suggests that zinc oxide nanoparticles promote cell elongation and thickening, which is crucial for the commercial quality of the fruits.

## 5. Conclusions

Foliar application of zinc oxide nanoparticles has a positive effect on chili fruit characteristics up to a concentration of 40 ppm, after which some parameters, such as fresh fruit weight and total fruit weight, decrease. The optimal concentration of GS-ZnONP suspension to improve the characteristics of chili fruits is 40 ppm, applied as a foliar spray every two weeks. At that ZnONP level, fruit size, mass, per plant production, vitamin C level, flavonoid content, antioxidant activity, Na^+^, Ca^+2^, Mg^+2^, Zn^+2^, and Mn^+2^ contents reached the highest level in the studied range, and other parameters increased considerably in comparison to the control.

Applying zinc oxide nanoparticles to chili plants favors seed germination [22,34], promotes plant growth and biomass production [67], and improves fruit quality. The treatments generate a greater concentration of phytochemical compounds such as phenols and flavonoids, as well as increased antioxidant activity and a higher concentration of essential minerals, which provide benefits for both agricultural crops and consumers. It is essential to emphasize that our results indicate that the application must be at appropriate concentrations to prevent phytotoxic effects and only benefit growing plants, thereby enhancing the nutritional value of the chili fruit. Thus, the foliar application of a correct concentration of ZnONPs has potential applications as a nanofertilizer and biostimulant, producing a nutrient-rich crop.

## Figures and Tables

**Table 1:** Effects of GS-ZnONPs on the diameter, length, fresh weight of fruit, number of fruits per plant, and total weight of the fruit obtained from each treatment.

GS-ZnONP Concentration (ppm)	Fruit Diameter (cm)	Fruit Length (cm)	Fresh Fruit Mass (g)	Number of Fruit per Plant	Total Mass of Fruit (g)
0	0.79 ± 0.17 d	8.97 ± 0.15 d	4.12 ± 0.17 c	292.75 ± 5.32 c	1047.65 ± 52 c
10	0.82 ± 0.05 c	9.52 ± 0.52 bc	4.76 ± 0.18 b	295.75 ± 22.14 b	1456.85 ± 29 b
20	0.87 ± 0.09 c	9.02 ± 0.73 c	5.06 ± 0.05 a	297.75 ± 2.31 bc	1486.01 ± 10 b
30	1.07 ± 0.14 b	10.42 ± 0.40 b	5.31 ± 0.33 a	347.50 ± 26.12 ab	1502.28 ± 48 b
40	1.22 ± 0.02 a	11.45 ± 0.17 a	5.35 ± 0.23 a	396.23 ± 5.20 a	2056.41 ± 31 a
50	1.12 ± 0.17 b	12.41 ± 0.35 a	4.01 ± 0.12 b	383.51 ± 13.21 a	1680.08 ± 15 ab

Values with different letters within the same column indicate significant differences according to the Tukey test (*p* ≤ 0.05). The values are the average of three repetitions ± standard deviation.

**Table 2:** Effects of GS-ZnONP concentration on vitamin C content in fruits of *Capsicum annuum*.

GS-ZnONP Concentration (ppm)	Vitamin C (mg 100 g^−1^ FW)
0	128.07 ± 4.15 c
10	131.21 ± 2.52 c
20	129.25 ± 3.73 c
30	141.42 ± 4.40 b
40	151.65 ± 3.17 a
50	112.41 ± 2.35 d

Values with different letters within the same column indicate a significant difference according to Tukey’s test (*p* ≤ 0.05). The values are the average of three repetitions ± standard deviation.

**Table 3:** Effects of GS-ZnONP concentration on macromineral content in *Capsicum annuum* fruit.

GS-ZnONPs (ppm)	Macro Minerals (g kg^−1^)					
	N	P	K	Ca	Na	Mg
0	13.44 ± 1.35 d	5.35 ± 0.11 d	4.28 ± 0.39 d	2.71 ± 0.18 b	0.13 ± 0.01 d	1.78 ± 0.01 c
10	14.54 ± 2.04 c	6.41 ± 0.12 b	6.98 ± 0.42 a	3.73 ± 0.19 a	0.18 ± 0.01 c	1.87 ± 0.03 b
20	15.72 ± 1.82 b	6.97 ± 0.09 a	5.35 ± 0.29 c	3.81 ± 0.23 a	0.20 ± 0.01 b	1.89 ± 0.01 b
30	16.79 ± 1.72 a	6.95 ± 0.04 a	5.98 ± 0.33 bc	3.99 ± 0.21 a	0.20 ± 0.01 b	1.94 ± 0.01 a
40	14.28 ± 1.10 c	5.90 ± 0.08 c	6.86 ± 0.32 b	3.51 ± 0.20 a	0.23 ± 0.02 a	1.92 ± 0.02 a
50	14.02 ± 0.93 cd	5.76 ± 0.17 c	5.32 ± 0.46 c	3.22 ± 0.10 ab	0.22 ± 0.03 a	1.71 ± 0.04 d

Values with different letters within the same column indicate a significant difference according to Tukey’s test (*p* ≤ 0.05). The values are the average of three repetitions ± standard deviation.

**Table 4:** Effects of GS-ZnONP concentration on micromineral content in *Capsicum annuum* fruit.

GS-ZnONPs (ppm)	Micro Minerals (mg kg^−1^)			
	Cu	Fe	Zn	Mn
0	13.01 ± 0.43 c	178.13 ± 1.21 d	25.27 ± 0.19 d	24.03 ± 0.12 c
10	12.21 ± 0.12 d	223.41 ± 1.26 c	85.21 ± 0.23 c	36.23 ± 0.38 bc
20	15.52 ± 0.23 a	248.45 ± 1.14 c	95.25 ± 0.45 b	40.78 ± 0.28 b
30	15.68 ± 0.31 a	779.67 ± 4.21 b	122.21 ± 1.02 a	41.02 ± 0.31 b
40	14.34 ± 0.25 b	825.62 ± 2.56 b	123.12 ± 1.02 a	52.34 ± 0.24 a
50	14.27 ± 0.19 b	1054.21 ± 2.23 a	97.12 ± 0.98 b	51.21 ± 0.32 a

Values with different letters within the same column indicate a significant difference according to Tukey’s test (*p* ≤ 0.05). The values are the average of three repetitions ± standard deviation.

## Data Availability

The data presented in this study are available on request from the corresponding author.

## References

[1] Maurya A.P., Chauhan J., Yadav D.K., Gangwar R., Maurya V.K. (2020). Nutraceuticals and Their Impact on Human Health.

[2] Jędrusek-Golińska A., Górecka D., Buchowski M., Wieczorowska-Tobis K., Gramza-Michałowska A., Szymandera-Buszka K. (2020). Recent progress in the use of functional foods for older adults: A narrative review. Compr. Rev. Food Sci. Food Saf..

[3] Saleh B.K., Omer A., Teweldemedhin B. (2018). Medicinal uses and health benefits of chili pepper (*Capsicum* spp.): A review. MOJ Food Process Technol..

[4] Olatunji T.L., Afolayan A.J. (2020). Comparison of nutritional, antioxidant vitamins and capsaicin contents in *Capsicum annuum* and *C. frutescens*. Int. J. Veg. Sci..

[5] Azlan A., Sultana S., Huei C.S., Razman M.R. (2022). Effects of Different Chili Pepper: A Review. Molecules.

[6] Javed R., Usman M., Yücesan B., Zia M., Gürel E. (2017). Effect of zinc oxide (ZnO) nanoparticles on physiology and steviol glycosides production in micropropagated shoots of Stevia rebaudiana Bertoni. Plant Physiol. Biochem..

[7] Cerqueira M.Â., Pinheiro A.C., Ramos O.L., Silva H., Bourbon A.I., Vicente A.A. (2017). Advances in Food Nanotechnology.

[8] Seleiman M.F., Almutairi K.F., Alotaibi M., Shami A., Alhammad B.A., Battaglia M.L. (2020). Nano-Fertilization as an Emerging Fertilization Technique: Why Can Modern Agriculture Benefit from Its Use?. Plants.

[9] Husen A., Iqbal M. (2019). Nanomaterials and Plant Potential.

[10] Singh A., Singh N.B., Afzal S., Singh T., Hussain I. (2018). Zinc oxide nanoparticles: A review of their biological synthesis, antimicrobial activity, uptake, translocation and biotransformation in plants. J. Mater. Sci..

[11] Saravanan M., Gopinath V., Chaurasia M.K., Syed A., Ameen F., Purushothaman N. (2018). Green synthesis of anisotropic zinc oxide nanoparticles with antibacterial and cytofriendly properties. Microb. Pathog..

[12] Zhang H., Chen S., Jia X., Huang Y., Ji R., Zhao L. (2021). Comparation of the phytotoxicity between chemically and green synthesized silver nanoparticles. Sci. Total Environ..

[13] García-López J.I., Niño-Medina G., Olivares-Sáenz E., Lira-Saldivar R.H., Barriga-Castro E.D., Vázquez-Alvarado R., Rodríguez-Salinas P.A., Zavala-García F. (2019). Foliar application of zinc oxide nanoparticles and zinc sulfate boosts the content of bioactive compounds in habanero peppers. Plants.

[14] Sharma D., Afzal S., Singh N.K. (2021). Nanopriming with phytosynthesized zinc oxide nanoparticles for promoting germination and starch metabolism in rice seeds. J. Biotechnol..

[15] Selim S., Saddiq A.A., Ashy R.A., Baghdadi A.M., Alzahrani A.J., Mostafa E.M., Al Jaouni S.K., Elamir M.Y.M., Amin M.A., Salah A.M. (2025). Bimetallic selenium/zinc oxide nanoparticles: Biological activity and plant biostimulant properties. AMB Express.

[16] Garza-Alonso C.A., Juárez-Maldonado A., González-Morales S., Cabrera-De la Fuente M., Cadenas-Pliego G., Morales-Díaz A.B., Trejo-Téllez L.I., Tortella G., Benavides-Mendoza A. (2023). ZnO nanoparticles as potential fertilizer and biostimulant for lettuce. Heliyon.

[17] Khan M.T., Ahmed S., Shah A.A., Noor Shah A., Tanveer M., El-Sheikh M.A., Siddiqui M.H. (2021). Influence of Zinc Oxide Nanoparticles to Regulate the Antioxidants Enzymes, Some Osmolytes and Agronomic Attributes in *Coriandrum sativum* L. Grown under Water Stress. Agronomy.

[18] Pejam F., Ardebili Z.O., Ladan-Moghadam A., Danaee E. (2021). Zinc oxide nanoparticles mediated substantial physiological and molecular changes in tomato. PLoS ONE.

[19] Suganya A., Saravanan A., Manivannan N. (2020). Role of Zinc Nutrition for Increasing Zinc Availability, Uptake, Yield, and Quality of Maize (*Zea mays* L.) Grains: An Overview. Commun. Soil Sci. Plant Anal..

[20] Asmat-Campos D., López-Medina E., Montes de Oca-Vásquez G., Gil-Rivero E., Delfín-Narciso D., Juárez-Cortijo L., Villena-Zapata L., Gurreonero-Fernández J., Rafael-Amaya R. (2022). ZnO Nanoparticles Obtained by Green Synthesis as an Alternative to Improve the Germination Characteristics of *L. esculentum*. Molecules.

[21] García-Gómez C., Obrador A., González D., Babín M., Fernández M.D. (2017). Comparative effect of ZnO NPs, ZnO bulk and ZnSO_4_ in the antioxidant defences of two plant species growing in two agricultural soils under greenhouse conditions. Sci. Total Environ..

[22] Sánchez-Pérez D.M., Flores-Loyola E., Márquez-Guerrero S.Y., Galindo-Guzman M., Marszalek J.E. (2023). Green Synthesis and Characterization of Zinc Oxide Nanoparticles Using Larrea tridentata Extract and Their Impact on the In-Vitro Germination and Seedling Growth of *Capsicum annuum*. Sustainability.

[23] Faizan M., Hayat S., Pichtel J., Hayat S., Pichtel J., Faizan M., Fariduddin Q. (2020). Effects of Zinc Oxide Nanoparticles on Crop Plants: A Perspective Analysis. Sustainable Agriculture Reviews 41.

[24] Rajput V.D., Minkina T., Fedorenko A., Chernikova N., Hassan T., Mandzhieva S., Sushkova S., Lysenko V., Soldatov M.A., Burachevskaya M. (2021). Effects of zinc oxide nanoparticles on physiological and anatomical indices in spring barley tissues. Nanomaterials.

[25] Pratap V., Durgesh K., Sheo M., Devendra K., Singh S., Singh V.P., Singh S., Tripathi D.K., Prasad S.M., Chauhan D.K. (2021). Plant Responses to Nanomaterials.

[26] Asghari M., Hasanlooe A.R. (2015). Interaction effects of salicylic acid and methyl jasmonate on total antioxidant content, catalase and peroxidase enzymes activity in “Sabrosa” strawberry fruit during storage. Sci. Hortic..

[27] Faizan M., Faraz A., Hayat S. (2020). Effective use of zinc oxide nanoparticles through root dipping on the performance of growth, quality, photosynthesis and antioxidant system in tomato. J. Plant Biochem. Biotechnol..

[28] Olatunbosun A., Nigar H., Rovshan K., Nurlan A., Boyukhanim J., Narmina A., Ibrahim A. (2023). Comparative impact of nanoparticles on salt resistance of wheat plants. MethodsX.

[29] Ahmed M., Tóth Z., Decsi K. (2024). The Impact of Salinity on Crop Yields and the Confrontational Behavior of Transcriptional Regulators, Nanoparticles, and Antioxidant Defensive Mechanisms under Stressful Conditions: A Review. Int. J. Mol. Sci..

[30] Khan S., Zahoor M., Sher Khan R., Ikram M., Islam N.U. (2023). The impact of silver nanoparticles on the growth of plants: The agriculture applications. Heliyon.

[31] Steiner A.A. (1961). A universal method for preparing nutrient solutions of a certain desired composition. Plant Soil.

[32] Hosen Z., Afroz Bipasha S., Kamal S., Rafique S., Islam B., Fatema K. (2020). Dietary Supplementation of *Citrus limon* L. (Lemon) and Evaluation of Its Role to Prevent and Cure of Vitamin C Deficiency Diseases. Int. J. Nutr. Food Sci..

[33] Lichtenthaler H.K. (1987). Chlorophylls and Carotenoids: Pigments of Photosynthetic Biomembranes. Methods Enzymol..

[34] Sánchez-Pérez D.M., Márquez-Guerrero S.Y., Ramírez-Moreno A., Rodríguez-Sifuentes L., Galindo-Guzmán M., Flores-Loyola E., Marszalek J.E. (2023). Impact of Biologically and Chemically Synthesized Zinc Oxide Nanoparticles on Seed Germination and Seedlings’ Growth. Horticulturae.

[35] Singleton V.L., Orthofer R., Lamuela-Raventós R.M. (1999). Analysis of total phenols and Other oxidation substrates and antioxidants by means of Folin-Ciocalteu Reagent. Sci. Hortic..

[36] Zhishen J., Mengcheng T., Jianming W. (1999). The determination of flavonoid contents in mulberry and their scavenging effects on superoxide radicals. Food Chem..

[37] Brand-Williams W., Cuvelier M.E., Berset C. (1995). Use of a free radical method to evaluate antioxidant activity. LWT-Food Sci. Technol..

[38] Collins M.D., Wasmund L.M., Bosland P.W. (1995). Improved method for quantifying capsaicinoids in *Capsicum* using high-performance liquid chromatography. HortScience.

[39] Ryu W.K., Kim H.W., Kim G.D., Rhee H.I. (2017). Rapid determination of capsaicinoids by colorimetric method. J. Food Drug Anal..

[40] Aebi H. (1984). Catalase in vitro. Methods Enzymol..

[41] Nelson D.P., Kiesow L.A. (1972). Enthalpy of decomposition of hydrogen peroxide by catalase at 25 °C (with molar extinction coefficients of H_2_O_2_ solutions in the UV). Anal. Biochem..

[42] Bradford M.M. (1976). A rapid and sensitive method for the quantitation of microgram quantities of protein utilizing the principle of protein-dye binding. Anal. Bio.

[43] Bremner J.M. (1960). Determination of nitrogen in soil by the Kjeldahl method. J. Agric. Sci..

[44] Oliveira J.P.S., Silva F.L.F., Monte R.J.G., Matos W.O., Lopes G.S. (2017). A new approach to mineralization of flaxseed (*Linum usitatissimum* L.) for trace element analysis by flame atomic absorption spectrometry. Food Chem..

[45] Tang S., Wang J., Zhu X., Shen D. (2024). Ecological Risks of Zinc Oxide Nanoparticles for Early Life Stages of Obscure Puffer (*Takifugu obscurus*). Toxics.

[46] Rossi L., Fedenia L.N., Sharifan H., Ma X., Lombardini L. (2019). Effects of foliar application of zinc sulfate and zinc nanoparticles in coffee (*Coffea arabica* L.) plants. Plant Physiol. Biochem..

[47] Amezcua J.C., Lara M. (2017). El Zinc en las Plantas. Ciencia.

[48] Gonmei G., Deb P., Kumar P., Sinha D., Halder A. (2022). Zinc Nutrition in Banana (cv. Grand Naine) at Early Growth Stage. Int. J. Plant Soil Sci..

[49] Jaithon T., Atichakaro T., Phonphoem W., T-Thienprasert J., Sreewongchai T., T-Thienprasert N.P. (2024). Potential usage of biosynthesized zinc oxide nanoparticles from mangosteen peel ethanol extract to inhibit *Xanthomonas oryzae* and promote rice growth. Heliyon.

[50] Ahmed R., Uddin M.K., Quddus M.A., Samad M.Y.A., Hossain M.A.M., Haque A.N.A. (2023). Impact of Foliar Application of Zinc and Zinc Oxide Nanoparticles on Growth, Yield, Nutrient Uptake and Quality of Tomato. Horticulturae.

[51] Siddiqui Z.A., Parveen A., Ahmad L., Hashem A. (2019). Effects of graphene oxide and zinc oxide nanoparticles on growth, chlorophyll, carotenoids, proline contents and diseases of carrot. Sci. Hortic..

[52] Chen J., Liu X., Wang C., Yin S.S., Li X.L., Hu W.J., Simon M., Shen Z.J., Xiao Q., Chu C.C. (2015). Nitric oxide ameliorates zinc oxide nanoparticles-induced phytotoxicity in rice seedlings. J. Hazard. Mater..

[53] García-López J., Zavala-García F., Olivares-Sáenz E., Lira-Saldívar R., Díaz Barriga-Castro E., Ruiz-Torres N., Ramos-Cortez E., Vázquez-Alvarado R., Niño-Medina G. (2018). Zinc Oxide Nanoparticles Boosts Phenolic Compounds and Antioxidant Activity of *Capsicum annuum* L. during Germination. Agronomy.

[54] He A., Jiang J., Ding J., Sheng G.D. (2021). Blocking effect of fullerene nanoparticles (nC_60_) on the plant cell structure and its phytotoxicity. Chemosphere.

[55] Lv Z., Sun H., Du W., Li R., Mao H., Kopittke P.M. (2021). Interaction of different-sized ZnO nanoparticles with maize (*Zea mays*): Accumulation, biotransformation and phytotoxicity. Sci. Total Environ..

[56] Schoofs H., Schmit J., Rink L. (2024). Zinc Toxicity: Understanding the Limits. Molecules.

[57] Sharma R., Bhardwaj R., Thukral A.K., Al-Huqail A.A., Siddiqui M.H., Ahmad P. (2019). Oxidative stress mitigation and initiation of antioxidant and osmoprotectant responses mediated by ascorbic acid in *Brassica juncea* L. subjected to copper (II) stress. Ecotoxicol. Environ. Saf..

[58] Mieszczakowska-Frąc M., Celejewska K., Płocharski W. (2021). Impact of innovative technologies on the content of vitamin C and its bioavailability from processed fruit and vegetable products. Antioxidants.

[59] Estevinho B.N., Carlan I., Blaga A., Rocha F. (2016). Soluble vitamins (vitamin B12 and vitamin C) microencapsulated with different biopolymers by a spray drying process. Powder Technol..

[60] Davarpanah S., Tehranifar A., Davarynejad G., Abadía J., Khorasani R. (2016). Effects of foliar applications of zinc and boron nano-fertilizers on pomegranate (*Punica granatum* cv. Ardestani) fruit yield and quality. Sci. Hortic..

[61] Kimura T., Kambe T. (2016). The functions of metallothionein and ZIP and ZnT transporters: An overview and perspective. Int. J. Mol. Sci..

[62] Balážová Ľ., Babula P., Baláž M., Bačkorová M., Bujňáková Z., Briančin J., Kurmanbayeva A., Sagi M. (2018). Zinc oxide nanoparticles phytotoxicity on halophyte from genus *Salicornia*. Plant Physiol. Biochem..

[63] Mahendra S., Zhu H., Colvin V.L., Alvarez P.J. (2008). Quantum dot weathering results in microbial toxicity. Environ. Sci. Technol..

[64] Tariverdizadeh N., Mohebodini M., Chamani E., Ebadi A. (2021). Iron and zinc oxide nanoparticles: An efficient elicitor to enhance trigonelline alkaloid production in hairy roots of fenugreek. Ind. Crops Prod..

[65] Pinedo-Guerrero Z.H., Delia Hernández-Fuentes A., Ortega-Ortiz H., Benavides-Mendoza A., Cadenas-Pliego G., Juárez-Maldonado A. (2017). Cu nanoparticles in hydrogels of chitosan-PVA affects the characteristics of post-harvest and bioactive compounds of jalapeño pepper. Molecules.

[66] Alvarez-Parrilla E., De La Rosa L.A., Amarowicz R., Shahidi F. (2011). Antioxidant activity of fresh and processed Jalapeño and Serrano peppers. J. Agric. Food Chem..

[67] Sanchez-Perez D.M., Flores-Loyola E., Orozco-Vidal J.A., Yescas-Coronado P., Rodriguez-Beltran R.I., Puente-Valenzuela C.O., Marszalek J.E., Marquez-Guerrero S.Y. (2025). The application of biosynthesized ZnO nanoparticles enhances the morphological and physiological indices of serrano pepper plants. Not. Bot. Horti Agrobot..

[68] Rai-Kalal P., Jajoo A. (2021). Priming with zinc oxide nanoparticles improve germination and photosynthetic performance in wheat. Plant Physiol. Biochem..

[69] Ruiz-Torres N., Flores-Naveda A., Barriga-Castro E.D., Camposeco-Montejo N., Ramírez-Barrón S., Borrego-Escalante F., Niño-Medina G., Hernández-Juárez A., Garza-Alonso C., Rodríguez-Salinas P. (2021). Zinc oxide nanoparticles and zinc sulfate impact physiological parameters and boosts lipid peroxidation in soil grown coriander plants (*Coriandrum sativum*). Molecules.

[70] Shehzadi N., Mahmood A., Kaleem M., Chishti M.S., Bashir H., Hashem A., Fathi E., Allah A., Shahid H., Ishtiaq A. (2024). Zinc and nitrogen mediate the regulation of growth, leading to the upregulation of antioxidant aptitude, physio-biochemical traits, and yield in wheat plants. Sci. Rep..

[71] Naziębło A., Bemowska-Kałabun O., Wierzbicka M., Zienkiewicz M. (2025). Foliar application of nitrates limits lead uptake by *Cucumis sativus* L. plants. J. Trace Elem. Med. Biol..

[72] Hernández-Pérez T., Gómez-García M.R., Valverde M.E., Paredes-López O. (2020). *Capsicum annuum* (hot pepper): An ancient Latin-American crop with outstanding bioactive compounds and nutraceutical potential. A review. Compr. Rev. Food Sci. Food Saf..

